# International Research Collaboration Supported by the U.S. National Institutes of Health: An Analysis of Coauthor Country Affiliations – 2014–23

**DOI:** 10.5334/aogh.5508

**Published:** 2026-10-16

**Authors:** Peter H. Kilmarx, Shirley Kyere

**Affiliations:** 1 Fogarty International Center, U.S. National Institutes of Health, Bethesda, Maryland, USA

**Keywords:** National Institutes of Health, international research collaboration, bibliometrics, scientific collaboration, research funding, citation impact, global health research

## Abstract

*Importance:* International collaboration is a critical component of biomedical and behavioral health research, but recent trends in its extent and impact in research funded by the U.S. National Institutes of Health (NIH), the world’s largest funder of health research, are not well characterized.

*Objective:* To characterize the extent and scientific impact of international collaboration in NIH-funded research from 2014 to 2023.

*Design, setting, and participants:* Bibliometric analysis of NIH-funded publications from 2014 to 2023 using the InCites Analytics database (Clarivate, London, UK).

*Main outcomes and measures:* Publication counts and author country affiliations for NIH-funded publications from 2014 to 2023 and scientific impact measured by Category Normalized Citation Impact (CNCI) for publications in 2023.

*Results:* The average annual number of NIH-funded publications from 2014 to 2023 was 112,633; this declined by 25.1% from 2021 to 2023. In 2023, 68.1% of publications had U.S.-affiliated authors only, 27.4% had both U.S.- and non–U.S.-affiliated authors, and 4.5% had non–U.S.-affiliated authors only. Overall, 31.9% of NIH-funded publications had non–U.S.-affiliated coauthors in 2023, a decline from a peak of 34.1% in 2018. Although CNCI values are not directly comparable across groups, publications with both U.S.- and non-U.S.-affiliated authors had a higher scientific impact (CNCI = 1.48) than those with U.S. authors only (1.32) or non-U.S. authors only (1.44).

*Conclusions and relevance:* International collaboration is a substantial component of NIH-supported research and is associated with higher citation impact. Nearly one-third of NIH-funded publications included non–U.S.-affiliated coauthors, and these publications demonstrated higher citation impact than those with U.S.-affiliated authors only, supporting the importance of global collaboration in biomedical research.

## Introduction

As a scientific agency and the world’s largest funder of biomedical and behavioral health research, the National Institutes of Health (NIH) has multiple compelling reasons for supporting international research collaborations. There are myriad research opportunities in unique populations around the world with differing genetic factors and disease presentations. There are also wide ranges in global environmental conditions, diets, infectious disease exposures, and cultural contexts, potentially yielding important scientific insights. International collaboration can harness perspectives from a broader range of investigators, impactful interventions, and alternative health systems. Global collaboration is particularly important for more timely and efficient completion of clinical trials, especially for those with more stringent eligibility criteria or for less common conditions.

The extent of international collaboration supported by the NIH can be assessed through bibliometric analysis of the affiliations of coauthors of publications that cite NIH funding support. An earlier study found that the percentage NIH-supported publications with non-U.S. affiliated coauthors rose from 29% in 2009 to 35% in 2017 [1]. By 2017, China had become the most frequently represented foreign country, with nearly 7000 NIH-supported publications with coauthors affiliated with China in that year.

Since 2017, several factors may have affected NIH-supported international research collaboration, including concerns about foreign influence and research security [2] and the impact of the COVID-19 pandemic [3]. In this study, we update the earlier analysis to characterize the extent, geographic distribution, and scientific impact of international collaboration in NIH-funded research from 2014 to 2023 in the context of these geopolitical changes.

## Methods

We analyzed data on 10 years of NIH-funded publications (2014–23) to identify the extent of international collaboration, geographic distribution, and scientific impact. Data were retrieved, analyzed, and visualized using the Web of Science Core Collection (Clarivate, London), InCites Benchmarking & Analytics (Clarivate, London, UK), Tableau (San Franciso, CA, USA), Datawrapper (Berlin, Germany), and Excel (Microsoft, Redmond, WA, USA). All data were accessed and exported on July 17, 2025.

To ensure precision and reproducibility, we used a two-step approach, first building curated groups using filters in Web of Science and InCites, then extracting document counts and Category Normalized Citation Impact (CNCI) using custom indicators. In step 1, publications were classified into three mutually exclusive categories. (1) “U.S.-Only Publications,” those with all coauthors with affiliations with institutions based only in the United States, were identified using the Locations Module, Funding Agency: National Institutes of Health (NIH) – USA, Metric: “Domestic Documents.” (2) “Non-U.S.-Only Publications,” those with no coauthors with affiliations with institutions based in the United States, were identified using the Funding Agencies Module, Funding Agency: NIH – USA, Researcher Location: Excluded = United States. And 3) “U.S. and Non-U.S. Publications,” those with coauthors with affiliations with institutions based in the United States AND with coauthors with affiliations with institutions not based in the United States, were identified using the Locations Module, Funding Agency: NIH – USA, Location Filter: “Collaborations with Locations: USA,” Baseline: All Items (for deduplication). In each case, the InCites Dataset was used, “Include ESCI documents” was checked, the metric was “Web of Science Documents,” and data were extracted separately for each year. For each group, the funding agency filters applied for all NIH Institutes and Centers, namely, CIT, CC, CSR, NICHD, FIC, NCCIH, NCI, NCATS, NEI, NHLBI, NHGRI, NIAID, NIAMS, NIBIB, NIDCR, NIDDK, NIEHS, NIGMS, NIMH, NINDS, NINR, NIA, NIAAA, NIDCD, NIDA, NIMHD, NLM, and the NIH OD.

In step 2, for each group and year, “Add Indicator” in InCites was used, “Category Normalized Citation Impact (CNCI)” was selected. CNCI is normalized by document type, subject area, and publication year. CNCI = 1.0 represents the global average; >1.0 indicates the above-average citation impact. It should be noted that CNCI values for analytic groups are calculated within separately normalized datasets and are not directly comparable as components of a single distribution.

We then created curated groups in Web of Science by manually selecting countries according to their World Bank income group and World Health Organization region classifications. These groups were then exported to InCites for analysis. Publications with only U.S.-affiliated authors were excluded from the analysis of publications in the high-income and Americas country groups. To visualize global publication output, we used Tableau (version 2025.2.1), Datawrapper, and Excel (version 2505). In the regional- and country-level analyses, publications were attributed to multiple regions or countries if there were individual coauthors with affiliations in more than one region or country or if there were different coauthors with affiliations in different regions or countries.

## Results

The number of publications citing NIH support increased from 104,827 in 2014 to 128,018 in 2021 and then decreased to 95,912 in 2023, a 25.1% decrease from the peak in 2021 (Figure 1). During the 10 years, the percentage of these publications with U.S.-affiliated authors only declined slightly from 69.9% in 2014 to a minimum of 65.9% in 2018 and then rose to 68.1% by 2023. The percentage with both U.S.-affiliated and non-U.S.-affiliated authors increased from 27.4% in 2014 to a maximum of 29.5% in 2018 and then declined to 27.4% in 2023. The percentage with non-U.S.-affiliated authors only increased from 2.7% in 2014 to a maximum of 5.6% in 2022 and then declined to 4.5% in 2023. The percentage of publications with any non-U.S. affiliated authors, the sum of the last two categories, ranged from 30.1% in 2014 to 34.1% in 2018 and then declined slightly to 31.9% in 2023. Overall, in this 10-year period, 368,102 publications funded by the NIH had a non-U.S. affiliated coauthor, 32.7% of the total. From the peak in 2021, the decline in NIH-funded publications with U.S.-affiliated authors only was 23.7%, while the decline in publications with any non-U.S.-affiliated authors was 27.8%.

**Figure 1:**
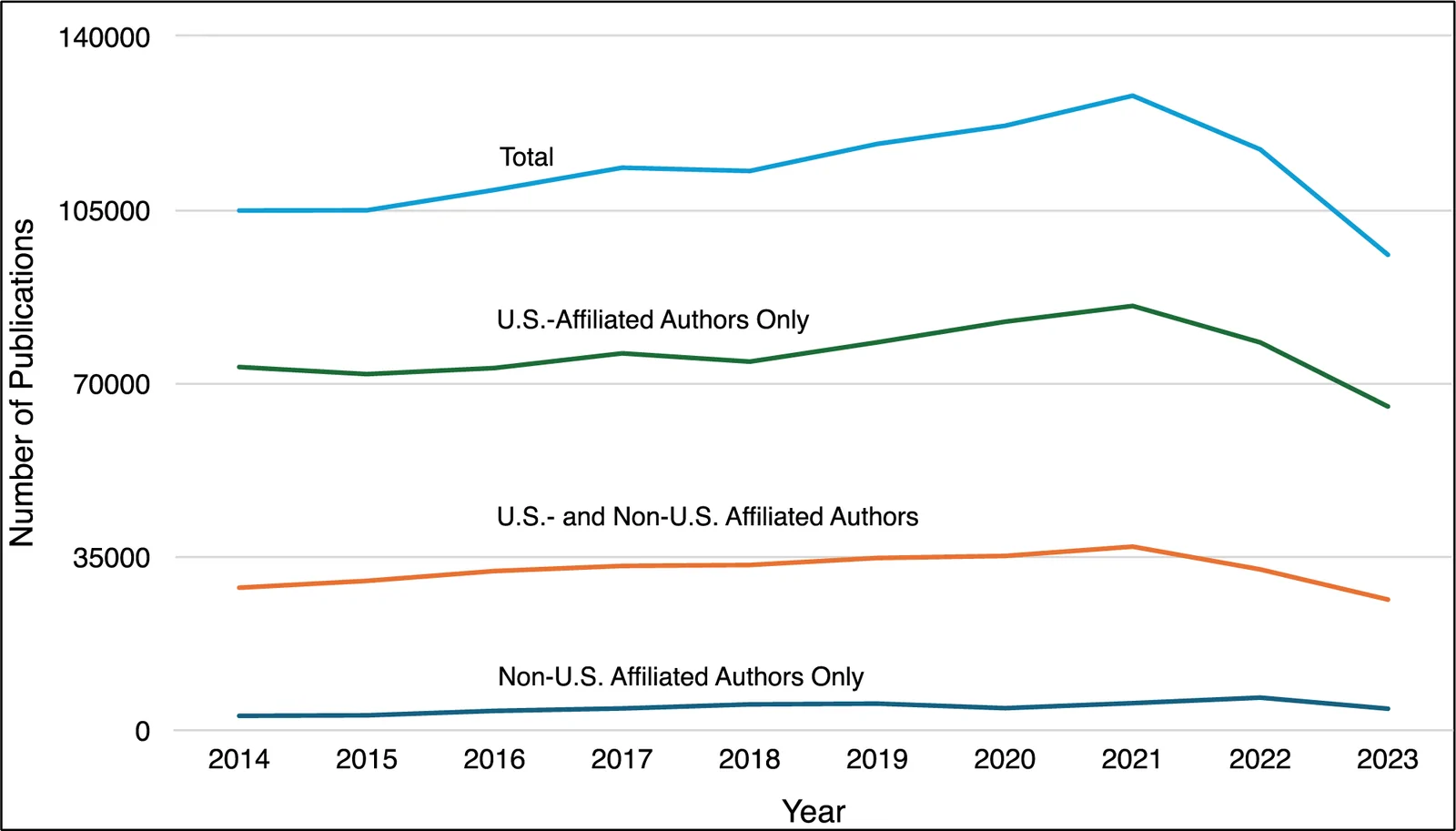
Number of publications funded by the U.S. National Institutes of Health by country of authors’ affiliations – 2014–23.

In 2023, the overall CNCI for the publications funded by NIH was 1.49, i.e., these studies were cited about one-and-a-half times as often as expected compared with similar publications (Table 1). The CNCI for NIH-funded publications with U.S.-affiliated authors only was lower (1.32) than those with both U.S.- and non-U.S.-affiliated authors (1.48) or publications with non-U.S.-affiliated authors only (1.44) (noting that CNCI values are not directly comparable across groups). By country income level, the CNCI was highest (2.06) for NIH-funded publications with coauthors with affiliations in high-income countries (not including publications with U.S.-affiliated authors only). By WHO region, the CNCI was highest for publications with coauthors with affiliations in the Eastern Mediterranean (2.22), Europe (2.21), and the Americas (not including publications with U.S.-affiliated authors only, 2.20).

**Table 1:** Publication count, scientific impact (Mean Category Normalized Citation Impact [CNCI]), and author order by author country affiliation of publications funded by the National Institutes of Health – 2023.

AUTHOR AFFILIATIONS	NUMBER OF PUBLICATIONS (% OF CATEGORY)	MEAN CATEGORY NORMALIZED CITATION IMPACT
All publications	95,881 (100%)	1.49
U.S. only	65,259 (68.1%)	1.32
U.S. and non-U.S.	26,324 (27.5%)	1.48
Non-U.S. only	4298 (4.5%)	1.44
**Author affiliations by country income level**		
Non-U.S. high-income countries	22,064 (67.1%)	2.06
Middle	9956 (30.3%)	1.82
Low	841 (2.6%)	1.73
**Author Affiliations by WHO region**		
Africa	2415 (6.6%)	1.55
Non-U.S. Americas	6704 (18.4%)	2.20
Eastern Mediterranean	1310 (3.6%)	2.22
Europe	14,948 (41.0%)	2.21
South-East Asia	1529 (4.2%)	2.15
Western Pacific	9569 (26.2%)	2.19

From 2014 to 2020, China was the most highly represented country for coauthor affiliations on NIH-funded publications (Figure 2A). By 2023, the United Kingdom was the highest (5129 NIH-funded publications), with Canada second (4677) and China third (4066) (Table 2). Other than China, the countries in the top 10 were high-income countries. In 2023, among the 10 most highly represented countries, the CNCI was highest for NIH-funded publications with coauthors with affiliations in Italy (3.39) and Spain (3.29) and lowest in China (2.31) (Table 2). The affiliation of the first author was from the foreign country most frequently for China (48.3%) and Japan (33.9%) and was under 30% of the publications from the other countries in the top 10. The affiliation of the last (senior) author was from that country most frequently for Australia (29.6%) and Canada (28.6%), and closer to 20% for most of the other countries in the top 10.

**Figure 2:**
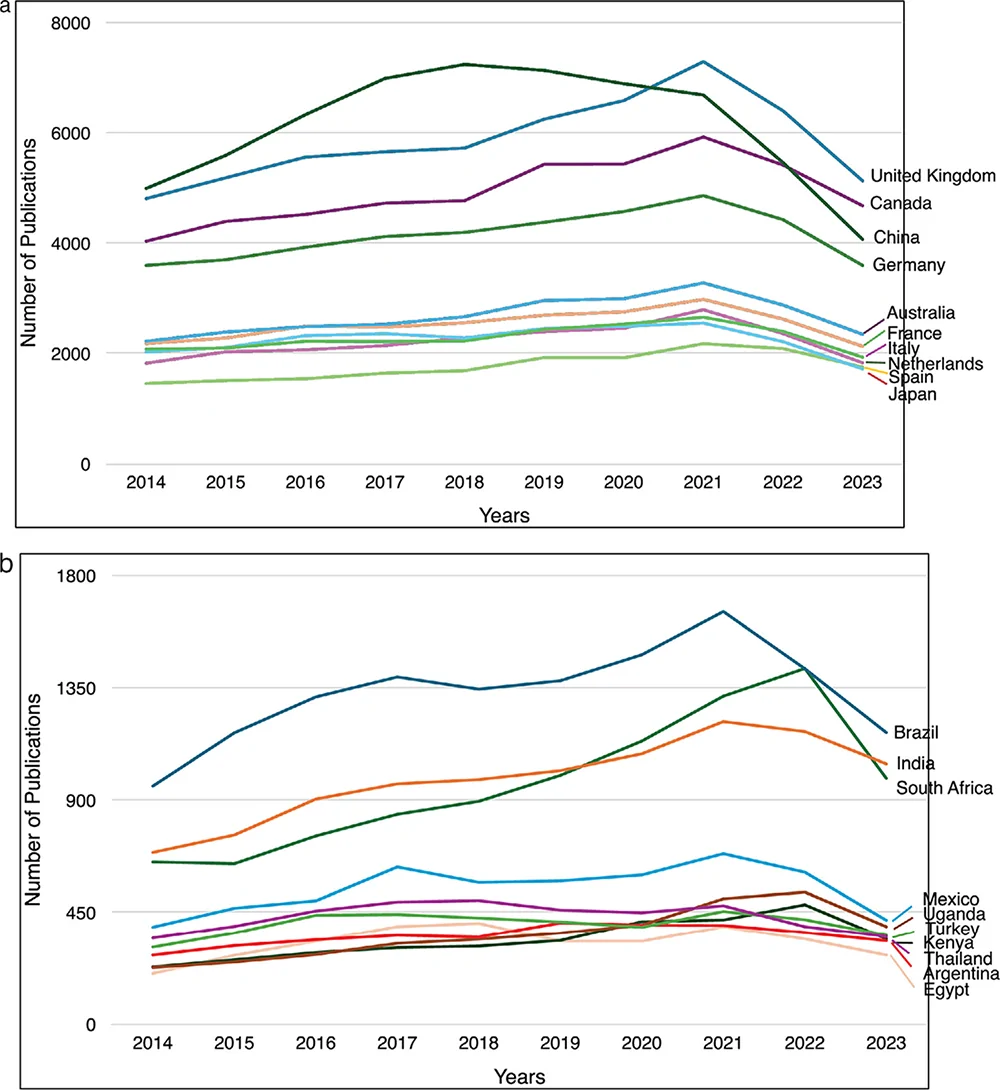
Number of Publications Funded by the U.S. National Institutes of Health by Foreign Country of Authors’ Affiliations – 2014–23. **(a)** Top 10 Countries; **(b)** Top 10 Low- and Middle-Income Countries (Excluding China).

**Table 2:** Publication count, scientific impact (Mean Category Normalized Citation Impact [CNCI]), and author order by author country affiliation of publications funded by the National Institutes of Health, top 10 countries overall and top 10 low- and middle-income countries (LMICs, other than China) by descending number of publications – 2022.

AUTHOR AFFILIATIONS	NUMBER OF PUBLICATIONS	MEAN CATEGORY NORMALIZED CITATION IMPACT	FIRST AUTHOR FROM COUNTRY	LAST AUTHOR FROM COUNTRY
United Kingdom	5129	2.81	28.8%	27.5%
Canada	4677	2.66	29.8%	28.6%
China	4066	2.31	48.3%	26.2%
Germany	3594	2.99	27.8%	23.0%
Australia	2344	2.97	29.7%	29.6%
France	2126	3.16	25.7%	22.0%
Italy	1929	3.39	29.7%	22.6%
Netherlands	1836	3.25	26.1%	21.5%
Spain	1750	3.32	27.5%	21.0%
Japan	1719	2.79	33.9%	22.2%
**Top 10 other LMICs**				
Brazil	1170	2.9	35.8%	26.7%
India	1045	2.45	36.9%	24.5%
South Africa	987	2.5	36.7%	39.4%
Mexico	418	3.77	26.1%	18.7%
Uganda	392	2.21	30.1%	26.8%
Turkey	358	4.03	30.7%	15.9%
Thailand	352	2.07	36.1%	20.7%
Kenya	343	2.27	21.3%	16.3%
Saudi Arabia	341	3.5	26.7%	13.2%
Russia	294	4.38	21.8%	21.75%

For low- and middle-income countries other than China during 2014–2020, Brazil was the most highly represented country (Figure 2B), with 1170 NIH-funded publications with one or more Brazil-affiliated coauthors in 2023, followed by India (1045) and South Africa (987) (Table 2). Among these countries in 2023, the highest CNCI was for publications with Russia-affiliated coauthors (4.38), followed by Turkey (4.03). The first author was affiliated with the country most frequently for India (36.9%), followed by South Africa (36.7%), and the last author was affiliated with the country most commonly for South Africa (39.4), followed by Uganda (26.8%).

A map of the quintiles of the number of NIH-funded publications by country of the coauthors’ affiliation shows the greatest numbers in North America, western Europe, and certain countries in South America, southern and eastern Africa, southern and eastern Asia, and Oceania, and relatively fewer publications in Central America, eastern Europe, other regions of Africa, and western and central Asia (Figure 3). The number of NIH-funded publications in 2023 and CNCI for those publications by country coauthor affiliation for all countries with population size greater than 100,000 is available in Supplemental File 1.

**Figure 3:**
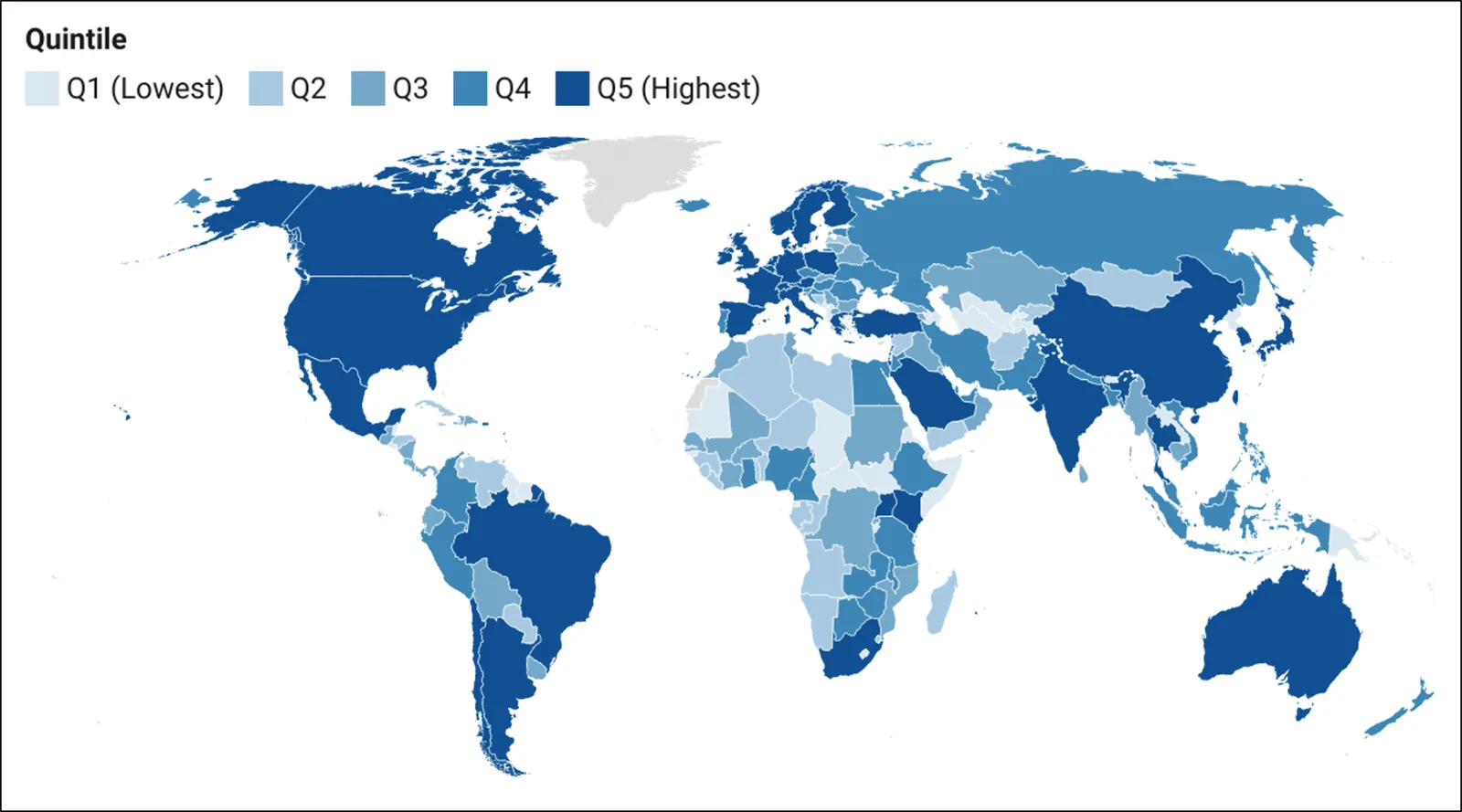
Quintiles of number of publications funded by the U.S. National Institutes of Health by country of authors’ affiliations – 2023.

## Discussion

We analyzed NIH-funded publications by coauthor country affiliation from 2014 to 2023 using the Web of Science database to characterize trends in NIH-supported international collaboration during a period marked by substantial global and policy changes. The proportion of publications with non–U.S.-affiliated coauthors increased from 30.1% in 2014 to a peak of 34.1% in 2018 and then declined modestly to 31.9% in 2023. This pattern was driven primarily by a decrease in publications with both U.S.- and non–U.S.-affiliated authors, which declined from 29.5% in 2018 to 27.4% in 2023, while publications with only non–U.S.-affiliated authors increased from 2.7% to 4.5% over the same period. Although these changes are modest, they suggest a leveling off and slight decline following the previously steady increase in international collaboration observed from 2009 to 2017 [1]. These trends occurred in the context of a broader decline in total NIH-funded publications after a peak in 2021; the number of publications with non–U.S.-affiliated coauthors declined by 27.8% from 2021 to 2023.

Despite these shifts, international collaboration remains a substantial component of NIH-supported research. Nearly one-third of NIH-funded publications included non–U.S.-affiliated coauthors, underscoring the extent to which biomedical research is a global enterprise. Publications involving international collaboration were associated with higher citation impact, as measured by CNCI, compared with those with U.S.-affiliated authors only. This finding is consistent with the potential for international collaboration to combine diverse populations, complementary expertise, and broader research perspectives, contributing to highly cited and influential scientific outputs.

NIH-supported international collaboration was concentrated in high-income countries, which accounted for approximately two-thirds of publications with non–U.S.-affiliated coauthors. Publications involving high-income country collaborators also had higher CNCI values than those involving low- and middle-income countries. This pattern may reflect both the characteristics of the CNCI metric, which tends to favor well-resourced research environments, and broader structural disparities in global research capacity and investment, as well as differential attention to diseases that disproportionately affect lower-income settings [4, 5, 6].

Several distinct but overlapping factors may explain the observed temporal trends. The overall increase in publications through 2021 followed by a marked decline through 2023 likely reflects the impact of the COVID-19 pandemic, including an initial surge in research output followed by downstream disruptions to laboratory and clinical research activities that reduced subsequent publication output. In contrast, the leveling and modest decline in the proportion of internationally collaborative publications beginning around 2018 predates the pandemic and may reflect broader structural or policy-related influences on international engagement. During the 2010s, strong interest in and support for global collaboration among investigators and NIH leadership likely contributed to increasing collaboration [7]. This trend may have reached a plateau in the late 2010s and may also have been influenced by changes in the global policy environment and U.S. research priorities affecting international engagement [8].

In particular, collaboration with China declined substantially after 2018, while collaboration with other high-income countries remained relatively stable. This pattern coincides with the implementation of the U.S. Department of Justice “China Initiative,” which focused on economic espionage and research security concerns [9], and is consistent with prior analyses documenting reductions in U.S.–China scientific collaboration during this period [2]. Since 2020, the COVID-19 pandemic likely further influenced patterns of collaboration through travel restrictions, disruptions to research infrastructure, and reduced opportunities to initiate new international partnerships. Together, these findings suggest that recent changes in NIH-supported research reflect both transient pandemic-related effects and longer-term geopolitical and policy dynamics that have altered the geographic distribution of international collaboration rather than leading to a uniform decline.

The patterns of authorship and citation impact varied across countries, suggesting differences in roles within international collaborations and research systems. Among the most highly represented countries, publications with coauthors affiliated with Italy and Spain had the highest CNCI, whereas those with China-affiliated coauthors had lower CNCI values. China and Japan also had the highest proportions of first authors affiliated with those countries, while senior (last) authorship was more frequently affiliated with countries such as Australia and Canada. These patterns may reflect differences in the structure of international collaborations, with some countries contributing more frequently to leading or primary analytic roles and others more commonly represented in senior or supervisory roles. In addition, variation in citation impact may reflect differences in research focus, journal selection, and integration into global scientific networks. These differences may also reflect variation in national research incentive structures and funding models that influence authorship roles and publication practices. Among low- and middle-income countries, there was similar heterogeneity in authorship patterns and CNCI, with relatively higher representation of first or senior authorship in countries such as India and South Africa, suggesting varying degrees of leadership within collaborative research. Together, these findings highlight the diversity of contributions to NIH-supported international research and the importance of considering authorship roles in understanding global scientific collaboration.

There are several limitations to this analysis. First, we analyzed author affiliations rather than nationalities; investigators may be affiliated with institutions outside their country of origin or residence, and publications with authors from multiple countries were attributed to each country represented, potentially overestimating geographic distribution. Second, identification of NIH-funded publications relied on funding acknowledgments in the Web of Science database, which may be incomplete or inconsistently reported. Third, CNCI is a normalized measure of citation impact but does not fully capture scientific or public health significance; as a mean of article-level scores, it may be influenced by a small number of highly cited publications. Also, CNCI values were calculated within InCites for separately defined analytic groups and are not directly comparable as components of a single distribution, which limits formal comparisons across groups. Fourth, publications involving international collaboration may differ systematically from U.S.-only publications in study scale, topic, or participation in large multicenter consortia; therefore, differences in CNCI should not be interpreted as causal effects of collaboration. Fifth, the Web of Science database may underrepresent certain regions or journals, particularly in low- and middle-income countries. Finally, there may be some lag in publication indexing, especially for international journals; we limited the analysis through 2023 to minimize this effect.

Recent policy changes at the NIH emphasize both the value and the risks of international research collaboration. The NIH Director’s statement on “Maximizing and Safeguarding NIH’s Investment in Foreign Collaborations” highlights a renewed focus on ensuring that international research delivers clear scientific and public value, particularly for the health of the U.S. population, while strengthening oversight and accountability [10]. These evolving policies reflect an effort to balance the benefits of global scientific engagement with concerns related to research security, transparency, and stewardship of public funds. The impact of these changes on the scale, structure, and outcomes of international collaboration remains to be seen. However, given the association observed in this analysis between international collaboration and higher scientific impact, it will be important to ensure that efforts to mitigate potential risks do not inadvertently diminish the scientific and public health benefits of global research partnerships. Ongoing evaluation will be needed to assess whether new policy approaches can successfully preserve the advantages of international collaboration while addressing legitimate concerns about oversight and accountability.

## Additional File

The additional file for this article can be found as follows:

Supplemental File 1. Excel file of number of NIH-funded publications with country coauthor affiliations and Category Normalized Citation Impact for those publications for countries with population size >100,000, by country – 2023.
